# Research Progress in the Treatment of Vaginitis With Bioactive Compounds: Targeting of Vaginal Microflora

**DOI:** 10.1002/mbo3.70203

**Published:** 2026-02-22

**Authors:** Yongming Li, Huiyu Liu, Ruihan Li, Rong Li, Jiaqi Liu

**Affiliations:** ^1^ Department of Gynecology Guigang Maternal and Child Health Care Hospital Guigang China; ^2^ College of Pharmacy Guangxi Medical University Nanning China; ^3^ Education Department of Guangxi Zhuang Autonomous Region, Key Laboratory of Environmental Pollution and Integrative Omics Guilin Medical University Guilin China; ^4^ State Key Laboratory of Quality Research in Chinese Medicine, School of Pharmacy Macau University of Science and Technology Macau China

**Keywords:** bioactive compounds, microflora, pharmacological activities, vaginitis

## Abstract

Vaginitis, a common gynecological ailment among women, poses a considerable health challenge with high recurrence rates even after conventional treatment. Vaginitis includes more common forms, such as bacterial vaginosis, vulvovaginal candidiasis, and *Trichomonas vaginalis* (TV), and less common forms, including senile vaginitis, infantile vaginitis, viral vaginitis, and mixed vaginitis. Conventional treatment approaches for vaginitis involve medications, such as metronidazole and clindamycin; however, long‐term use of these agents raises concerns about adverse reactions, disruption of the vaginal ecosystem, and antibiotic resistance. Bioactive compounds from Traditional Chinese Medicine (TCM), known for their lower toxicity and multifaceted biological activity, are effective for treating vaginitis with fewer side effects and a focus on restoring a healthy vaginal microecology. TCM decoctions exhibit internal effectiveness, while local TCM treatments utilize external medications and modalities, such as irrigation and fumigation sitz baths. This review offers insights to guide vaginitis treatment and provides a theoretical foundation for the development and application of herbal therapies for vaginitis.

## Graphical abstract

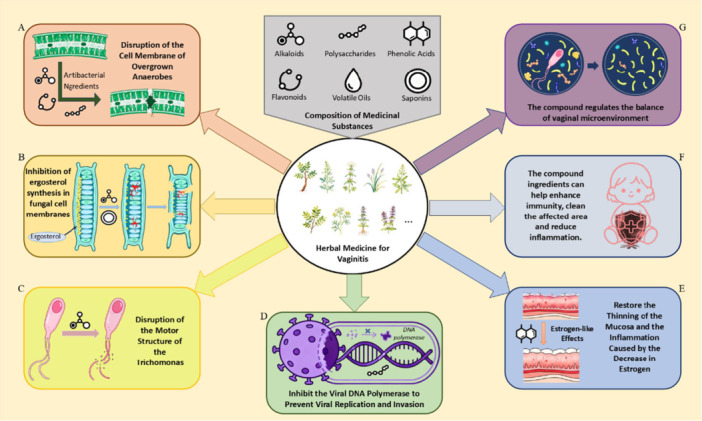

## 1 Introduction

**Figure 1:**
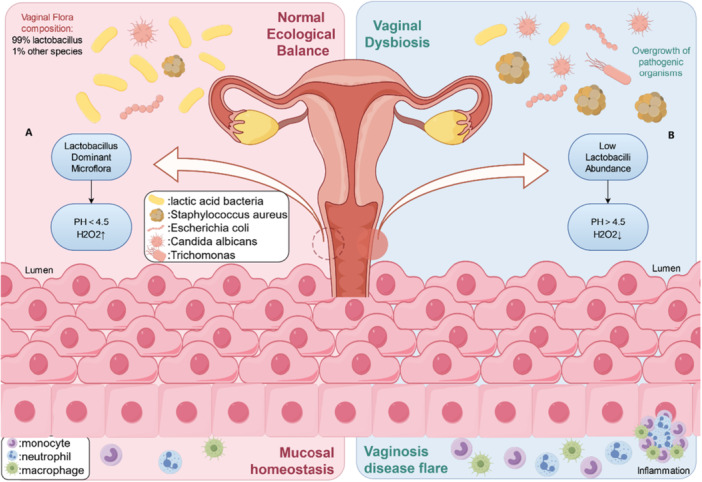
Vaginal microecology in normal and disturbed conditions. (A) Normal vaginal microenvironment dominated by *Lactobacillus*, when vaginal pH < 4.5 and H_2_O_2_ level is high. (B) The invasion of pathogenic bacteria into the vaginal microenvironment, the dominance of dominant bacteria in the vagina is plundered, resulting in the increase of vaginal pH, the decrease of H_2_O_2_ level, and the breakdown of the defense system of the vaginal wall, thus triggering an inflammatory response.

**Figure 2:**
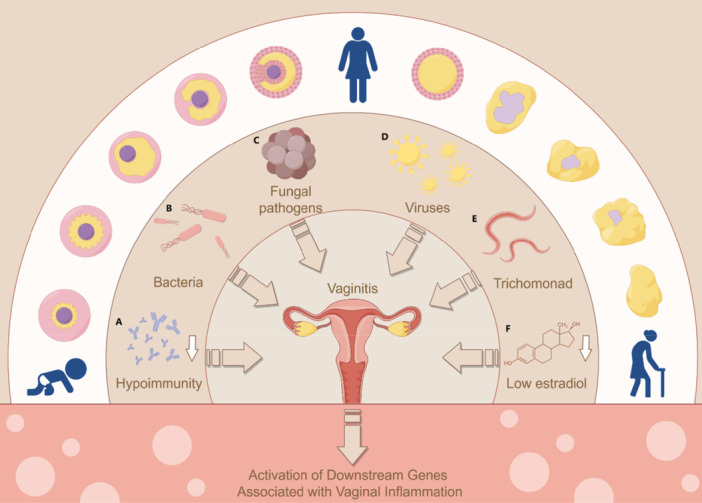
Causative factors for vaginitis. Women can be infected with vaginitis throughout their life, such as (A) infantile vaginitis due to weakened immunity, (B) bacterial vaginitis due to bacterial infection, (C) fungal infection of *Candida* vaginitis, (D) viruses infection of viral vaginitis, (E) Trichomonad infection with *Trichomonas* vaginitis, and (F) low estradiol levels of senile vaginitis.

Vaginitis, or vaginal inflammation, is associated with a variety of uncomfortable symptoms, including abnormal vaginal discharge, itching, irritation, and an unpleasant odor. Vaginitis is one of the most common diseases in women and leads to a wide array of health issues that can significantly impact a woman's quality of life. The most common forms of vaginitis include bacterial vaginosis (BV), vulvovaginal candidiasis (VVC), and *Trichomonas vaginalis* (TV) (Figure 1). Data shows that BV accounts for approximately 45%–55% of identified vaginitis cases (Lin et al. 2019). A survey conducted by the World HealthOrganization estimates that 75% of women experience symptoms caused by a *Candida* infection at least once in their lifetimes, and approximately 5%–10% of women with a primary VVC will develop recurrent VVC (Sherrard et al. 2018). Trichomoniasis, a sexually transmitted infection caused by the parasite *T. vaginalis*, accounts for 15%–20% of vaginitis cases (Schwebke and Burgess 2004). These types of vaginitis are usually treated with medications, including oral and/or intravaginal metronidazole, clindamycin, and other antibiotics (Paladine and Desai 2018). However, the long‐term and frequent use of antibiotics and probiotics can lead to serious adverse reactions, imbalances in the vaginal ecosystem, and increases in antibiotic resistance (Workowski et al. 2021). Moreover, currently available oral and vaginal probiotics exhibit limited efficacy for treating vaginitis (Marnach et al. 2022). Thus, it is essential to optimize the therapeutic strategies for vaginitis. Estrogen is an important factor affecting the vaginal microecology and vaginal epithelial structure of women. Additionally, the vaginal microbiota of women can shift during different physiological stages. For example, newborn female infants are in a state of estrogen silencing and immunocompromise, increasing their susceptibility to infantile vaginitis (Figure 2). During puberty and the childbearing years, follicular development leads to estrogen production. In healthy women of childbearing age, the vaginal microbiota is dominated by *Lactobacillus* species. Lactobacilli produce a large amount of lactic acid to create a vaginal pH of < 4.5, which protects the health of the vaginal microenvironment in certain scenarios, such as if there is an excessive amount of opportunistic pathogens causing microecological disorders and inflammation. In contrast, postmenopausal women are prone to developing senile vaginitis, which is caused by a lack of estrogen, decreasing levels of glycogen, and a thinning of the vaginal epithelium. In these women, the vaginal microenvironment is like that of the prepubertal vagina, increasing the likelihood of developing senile vaginitis (Kalia et al. 2020). Internal lesions caused by vaginitis are usually the result of multiple factors acting together. For instance, hormonal level changes and immune system dysfunction (Paladine and Desai 2018). Traditional Chinese Medicine (TCM) has significant advantages for treating vaginitis because it involves multiple components, targets, links, and pathways (R. Zhang et al. 2019). TCM is often considered to be less toxic and biologically active, and it has been invaluable in the search for novel antifungal drugs. Furthermore, TCM provides relatively effective treatments for vaginitis, with fewer side effects and better patient tolerance. TCM decoction treatments mostly involve medications that invigorate the spleen, support qi, regulate the liver and spleen, warm the kidneys and spleen, clear heat, and eliminate dampness. For instance, Wu Mei Yin Chen Tang, a prescription consisting of *Prunus mume*, *Zanthoxylum bungeanum*, *Lonicerae japonicae flos*, *Artemisia argyi*, *Polygonum hydropiper*, *Poria cocos*, *Atractylodes macrocephala*, *Phellodendri chinensis cortex*, and *Gentiana scabra*, is an effective internal treatment for vaginitis (Du et al. 2002). In contrast, local treatments involve the use of external medications around the vagina and certain treatment modalities, such as irrigation, fumigation sitz baths, and vaginal suppositories. Topical administration has several advantages, including its avoidance of potential gastrointestinal tract and liver damage and its bypass of the metabolic functions of these organs, thereby increasing medication effectiveness and maintaining drug concentrations in the blood (C. Liu et al. 2014). Different topical forms of TCM, including Cnidii Fructus, *Sophora flavescens*, Kochiae Fructus, *G. scabra*, and Coptidis Rhizoma, possess properties that clear heat and dampness, eradicate parasites, and relieve itching, thereby enhancing the body's immune capabilities. This review is focused on integrating the concepts of TCM into the treatment of vaginitis and discussing the current progress of treating vaginitis with commonly used Chinese medicines and acupuncture. In addition, we conclude with the functions and mechanisms of TCM for treating vaginitis, providing new insights into clinical vaginitis therapies.

## 2 Bacterial Vaginosis

### 2.1 BV Pathogenesis

The human vaginal microbiota consists of a complex ecosystem of beneficial microorganisms and potentially pathogenic bacteria, all of which coexist in the vaginal environment (Smith and Ravel 2017). Lactobacilli, the predominant microorganisms, generally account for over 95% of all vaginal bacteria and maintain an acidic environment, with a pH value ranging from 3.8 to 4.4 (Hong et al. 2022). These organisms secrete the enzyme responsible for breaking down glycogen into smaller glucose polymers, which are subsequently transported into the cell and fermented through pyruvate (Pyr). This process results in the production of lactic acid isomers, including both d‐lactic acid and l‐lactic acid, effectively acidifying the microenvironment to maintain microbial balance (Ilhan et al. 2019; Łaniewski et al. 2020). Furthermore, the breakdown products of glycogen can serve as a source for the generation of hydrogen peroxide (H_2_O_2_). The combination of a low vaginal pH and the presence of certain bacterial products, including lactic acid, bacteriocins, and H_2_O_2_, collectively acts to suppress the growth of less beneficial bacteria (Onderdonk et al. 2016).

A decrease in the number of lactobacilli and an increase in the proportion of aerobic bacteria, including *Escherichia coli*, *Staphylococcus aureus*, *Klebsiella*, and *Enterococcus*, induce an immune response, thus resulting in BV (Y. Liu et al. 2023). BV is a clinical condition characterized by a shift from a *Lactobacillus*‐dominated flora to a more complex flora dominated by strict and facultative anaerobic bacteria, including *Gardnerella vaginalis* (Mårdh 1993).

The diagnosis of BV is based on Amsel's criteria, which require the presence of at least three of the four following components: a thin and uniform white discharge, an elevated pH of > 4.5 in the vaginal fluid, the emission of an amine odor when potassium hydroxide is applied (yielding a positive whiff test), and the identification of more than 20% of clue cells on saline microscopy (Neal et al. 2020).

Recent guidelines from the Centers for Disease Control and Prevention in the United States recommend the use of either oral or intravaginal metronidazole and clindamycin for BV treatment. Oral or vaginal administration of probiotics containing *Lactobacillus* can be an effective adjunctive therapy for BV and other infectious symptoms (Bohbot et al. 2018; Recine et al. 2016). However, one study reported that 20%–75% of women experienced a recurrence of BV within 3 months after antibiotic treatment, indicating a high recurrence rate and an urgent need to develop new treatment strategies for the prevention and treatment of BV (Bradshaw et al. 2006). A randomized‐controlled trial (RCT) showed that *Lactobacillus* and probiotics serving as adjunctive therapies did not improve the cure rate of BV in Chinese patients (Y. Zhang et al. 2021). TCM, a broad therapeutic approach employing a more holistic perspective, is commonly used for BV treatment with minimal risk of drug resistance or side effects (Z. Wang et al. 2019).

### 2.2 Herbal Medicine for BV

TCM formulations have gained widespread recognition for their use in treating clinical BV due to their multifaceted abilities, including antibacterial effects, augmentation of biofilm permeability and drug susceptibility, immune system enhancement, and estrogen‐like impacts (Y. Zhang et al. 2022). TCM has evolved three distinct strategies for BV treatment: invigorating the spleen, clearing dampness and heat, and nourishing the kidneys (H. Zhao et al. 2021). Yihuang Decoction (YHD) has been widely used for treating conditions that are characterized by abnormal vaginal discharge through a spleen‐invigorating approach (X. Q. Wang et al. 2020). The efficacy of YHD is attributed to its core components, including Rhizoma Dioscoreae, Semen Euryales, *A. macrocephalae*, and *P. chinensis*. Polysaccharides derived from Rhizoma Dioscoreae have been shown to exhibit potent immune capabilities for spleen‐deficiency syndromes, as demonstrated by improved spleen pathology and increased levels of immune‐active substances (R. Huang et al. 2020). According to TCM principles, Semen Euryales plays a complementary role to Rhizoma Dioscoreae for spleen invigoration. In addition, *A. macrocephalae* polysaccharides have been shown to promote T‐lymphocyte proliferation, increase the percentages of CD4^+^ and CD8^+^ T cells, and primarily activate macrophages through the nuclear factor‐kappa B and Janus kinase‐signal transducer pathways (W. Sun et al. 2015; Xu et al. 2020). Similarly, Semen Plantaginis has been shown to have positive effects on immune pathways (Ji et al. 2019). Jiang et al. (2018) demonstrated that Semen Plantaginis polysaccharides effectively promote the maturation of dendritic cells from bone marrow by activating the mitogen‐activated protein kinase (MAPK) pathway, which is essential for both innate and adaptive pathways. These results suggest that the immunoenhancement effects of YHD occur through the regulation of T cells, macrophages, and dendritic cells in the spleen, which may be the fundamental therapeutic mechanism of this agent for BV treatment.

In modern medical theory, BV is highly associated with an invasion of external pathogenic factors (e.g., dampness and heat) into the lower part of the body. Bazhengsan, a traditional remedy historically employed for treating morbid leucorrhea attributed to dampness and heat, consists of several herbs, including Herba Dianthi, *Glycyrrhiza*, and matrine. In a clinical trial of metronidazole‐ and Bazhengsan‐treatment groups, significant improvements were reported in many vaginitis indicators, including vaginal discharge color, odor, viscosity, pruritus vulvae, vaginal microenvironment, and TCM syndrome (Y. F. Chen 2016). Bazhengsan exhibited a lower recurrence rate and superior results than metronidazole treatment (Y. F. Chen 2016). Herba Dianthi has been shown to possess antibacterial activity against both Gram‐negative bacteria (*Bacterium dysenteriae* and *Vibrio cholerae*) and Gram‐positive bacteria (S. Zhang et al. 2018). Furthermore, *Glycyrrhiza* has been shown to inhibit the growth of bacteria, including *S. aureus* (Ostafiichuk et al. 2022). Moreover, matrine has been shown to inhibit biofilm formation by antimicrobial‐resistant *E. coli* strains, which is a pivotal part of developing antibacterial resistance (Y. Sun et al. 2019). Of note, matrine had negligible effects on the growth of common vaginal *Lactobacillus* species, including *L. rhamnosus*, *L. crispatus*, *L. jensenii*, *L. gasseri*, *L. reuteri*, and *L. vaginalis*, at clinical dosages, indicating that it can inhibit aerobic bacterial growth and proliferation without harming native lactobacilli (Tao et al. 2019).

Numerous studies have reported that herbal medicines with strong antibacterial and anti‐inflammatory properties can serve as topical vaginal treatments. Cortex *P. chinensis* is a commonly used TCM for treating BV, both orally and topically. This formulation contains berberine, which has been shown to reduce oxidative stress by decreasing reactive oxygen species (ROS) and promoting the expression of superoxide dismutase (Ma et al. 2019). Berberine has been shown to inhibit apoptosis in vaginal epithelial cells by decreasing the expression levels of several apoptotic proteins, including caspase‐3, cytochrome C, caspase‐12, and Bcl‐2‐associated X‐protein (Ma et al. 2019). These clinical and laboratory findings illustrate the effectiveness of topical TCM treatments for BV.

### 2.3 Combination of Chinese and Western Medicines for the Treatment of BV

Current treatment approaches for BV consist primarily of medications targeting aerobic bacteria, probiotic therapy, and antibiotic treatment. Appropriate antibiotics include clindamycin, nifuratel, and cefuroxime, all of which have demonstrated good therapeutic effects when used in combination with TCM (Romanik et al. 2007). Liuwei Dihuang Decoction (LDD) and Zhibai Dihuang Decoction (ZDD) are TCM formulations that specialize in the treatment of BV attributable to a kidney deficiency coupled with damp‐heat syndrome. LDD has been shown to enhance the efficacy rate of tinidazole from 84.85% to 96.97%, while ZDD enhanced the efficacy rate of metronidazole treatment from 78.95% to 94.59% (Gong 2019; S. Yang et al. 2020). Combination therapy with ZDD exhibited a more extended effect, with only a 10% recurrence rate, while the recurrence rate was 22.5% for the control group after 3 months (Gong 2019). Similarly, a clinical RCT reported that the total efficacy rate of Longdan Xiegan Decoction (LXD), a compound TCM, reached 96.88% when combined with Western medicine, significantly surpassing the metronidazole suppository control group (84.88%) (Cui 2018). LXD has been shown to have both antibacterial and anti‐inflammatory properties and promote diuresis (Cui 2018). In these TCM concoctions, *Radix Rehmanniae* serves as the principal herb and functions by regulating estrogen levels and exhibiting estrogen‐like activity (Luo et al. 2020). Y. Y. Zhao et al. (2019) demonstrated that rehmapicrogenin, an active component of *Radix Rehmanniae*, contributes to its estrogenic effects by interacting with the estrogen receptors (ERs) G protein‐coupled receptor 30 and ERα, leading to the proliferation of MCF‐7 cells in vitro and increasing uterine development in immature female mice. The mechanisms by which TCM treats BV are largely related to its herbal components with kidney‐nourishing properties, which exhibit estrogenic activity.

Sophora alkaloids have become a valuable component of vaginal gels for BV treatment. The combination of metronidazole and Sophora‐containing washes has been shown to significantly improve BV and exhibit superior long‐term effects than metronidazole alone (Liang et al. 2019). This improvement has been attributed to the downregulation of inflammatory cytokines, including interleukin (IL)‐4, IL‐12, and interferon (IFN)‐γ, and an enhancement of the vaginal microenvironment (Liang et al. 2019). Moreover, Sophora alkaloid gel with nifuratel capsules has been shown to have a high overall efficacy rate, with reduced levels of inflammatory factors and serum oxidative stress (Y. Li et al. 2020). One study observed that, in 80 patients aged 20–55 years, the treatment of aerobic vaginitis and BV with clindamycin and a Fufang Furong Effervescent Suppository led to increased lactobacilli abundance and decreased levels of pathogenic bacteria (M. Li et al. 2022). Fufang Furong suppositories have been shown to have the same efficacy as clindamycin but are more effective at restoring vaginal flora. Similarly, a combination of a compound sea buckthorn seed oil suppository and metronidazole has been shown to significantly improve clinical symptoms and exhibit a better effect than metronidazole treatment alone (C. X. Hu 2020). Sea buckthorn seed oil is rich in sugar substances, which provide nutrients for the growth of dominant bacteria. The total sea buckthorn flavonoids contained in sea buckthorn seeds have antioxidant effects, which inhibit bacterial reproduction and promote tissue regeneration. Serpentine and bitter saxifrage have been shown to inhibit the growth and reproduction of pathogenic bacteria and to downregulate the levels of inflammatory factors to enhance immunity and improve the microecological environment of the vagina (D. Zhang et al. 2016). Fructus Hippophae is rich in total polyphenols and flavonoids, which may impact BV treatment through its regulation of serum estradiol levels (Criste et al. 2020; C. X. Hu 2020). These TCM combination therapies can improve abnormal blood levels of sex hormones and significantly elevate antibacterial activity, setting them apart as promising potential strategies for BV treatment.

## 3 Vulvovaginal Candidiasis

### 3.1 Pharmacologic Mechanisms for the Treatment of VVC

*Candida albicans* (*C. albicans*) is one of the most common human fungal pathogens to cause mucosal and systemic infections. *C. albicans* is mainly observed in the mucous membranes of the skin, oral cavity, gastrointestinal tract, and genital tract (Tasaki et al. 2018). VVC is a common gynecological fungal infectious disease caused by *C. albicans* infection and characterized by a colonization of the vaginal mucosa (De Bernardis et al. 2018). More than 75% of women will develop VVC at least once in their lives, and 5%–10% of these women will have a high recurrence rate of VVC (more than four episodes per year) (Zahedi et al. 2016). Patients with VCC can experience uncomfortable symptoms, such as vulvar itching, dyspareunia, dysuria, a burning sensation, and a curd‐like discharge (L. Chen et al. 2022).

The laboratory diagnosis of VVC is based on Cibley's criteria, which involve the absence of *Trichomonas*, *Gardnerella*, or *Candida*; an elevated quantity of lactobacilli (often adherent to intermediate epithelial cells); a scarcity of white cells; proof of cytolysis with bare or naked intermediate nuclei, and a pH ranging from 3.5 to 4.5 (Qi et al. 2021).

Clinical treatment of VVC mainly relies on azole antifungal drugs, including fluconazole. These drugs inhibit the synthesis pathway of ergosterol on the fungal cell membrane, thereby inhibiting fungal growth. However, the presence of a *C. albicans* biofilm can bolster its drug resistance and virulence. The phenomenon of drug resistance in certain strains is dramatically increasing and causes damage to normal vaginal cells and tissues, exacerbating imbalances in the local vaginal microbiota, creating acid–base balance disorders, and leading to repetitive infections, thereby significantly increasing the difficulty of clinical treatment with long‐term extensive use (Wei et al. 2023).

### 3.2 Topical Application for the Treatment of VVC

Local treatment of VVC mainly involves the use of external medications on the outside of the vagina, with the main treatment modalities involving irrigation, fumigation sitz baths, and vaginal suppositories. Studies have reported that colonization by *C. albicans* recruits many neutrophils into the vagina, which leads to massive collateral damage, further prolonging the activation of immune cells and, ultimately, organ dysfunction (Brandes et al. 2013). Perillaldehyde, a traditional antifungal drug extracted from *Perilla frutescens*, reverses the host damage caused by a hyperactive immune response in the vagina. After treatment with this agent, the amount of neutrophils in tissues is significantly decreased, accompanied by a gradual recovery of tissue integrity (Bao et al. 2023). *C. albicans* induces a large number of macrophages to aggregate in the vagina, and perillaldehyde treatment has been shown to reduce the concentration of tumor necrosis factor (TNF)‐α, a protein mainly secreted by macrophages, to normal levels (Qu et al. 2019). Moreover, perillaldehyde has been shown to inhibit several virulence attributes of *C. albicans*, including biofilm formation, yeast‐to‐hyphal transition, and secreted aspartic proteinases gene expression (Qu et al. 2019). Thus, perillaldehyde provides a new strategy for clinical antifungal treatment.

Baofukang suppositories have been shown to effectively inhibit *Candida* vaginal adhesion, hyphal formation, and proliferation, leading to a restoration of vaginal epithelial cell morphology and vitality, along with an enhancement of local immune function (T. Li et al. 2016). These suppositories have been shown to exhibit significant antifungal properties, potentially achieved through upregulation of Type 1 helper (Th1) cell immunity, bolstering the Th17 axis of the innate immune response, and promoting the secretion of immunoglobulin G derived from the vaginal epithelium (T. Li et al. 2016). In addition, the Huo Xiang washing lotion compound has been shown to exhibit marked efficacy for curbing pathogenic fungi, leading to rapid relief from symptoms such as itching, exudation, and erosion that are commonly associated with fungal infections when used in combination with Western medicine or alone (Na et al. 2003). Similarly, the cure rate, the negative‐to‐positive ratio of fungi, the efficacy rate, and the recurrence rate of miconazole combined with Honghe washing solution were superior to miconazole alone for the treatment of VVC (Lijuan et al. 2022). These combined effects collectively restore immune function to better protect against *C. albicans*.

### 3.3 Modulation of Immunosuppression of VVC by TCM

Baitouweng Decoction, first prescribed about 1800 years ago, contains four herbal plants: *Pulsatilla chinensis*, Golden cypress, *Coptis chinensis*, and *Cortex fraxini*. In a clinical trial of 49 patients with recurrent VVC, the recurrence rate of the Baitouweng Tang treatment group was 25%, while the recurrence rate of the fluconazole control group was 47%, indicating that long‐term administration of Baitouweng Decoction is significantly more effective than standard chemical treatments (Zhang and Zhang 2012). In mice, an *n*‐butanol extract derived from Baitouweng Decoction induced a decrease in fungal load and *C. albicans* hyphal count in vaginal secretions, with reduced levels of several inflammatory markers, including TNF‐α, IL‐1β, IL‐18, and lactate dehydrogenase (Zhang and Zhang 2012), and its potential treatment mechanism may be linked to activation of the PKCδ/NLRC4/IL‐1Ra axis, resulting in the downregulation of *NLRP3* inflammasome expression, consequently inhibiting the production of the inflammatory factors IL‐1β and IL‐18 (Zhang and Zhang 2012). *Pulsatilla chinensis* is rich in pentacyclic triterpenoid saponins, and these saponins have been shown to interact with the biofilm layer to disrupt the membrane, thereby leading to perforation or complete membrane lysis (Zhong et al. 2022). Dioscin, a natural steroid saponin, has been shown to inhibit biofilm formation, the development of *C. albicans*, morphological transformation, adhesion, and other virulence factors (L. Liu et al. 2015). Dioscin exerts considerable antifungal activity by disrupting the structure of fungal membranes after invasion, resulting in fungal cell death. Dectin‐1, a primary pattern‐recognition receptor identifying β‐glucan, has been linked to the heightened susceptibility of Dectin‐1 mice to *C. albicans* infections, which is caused by compromised cytokine generation and reduced neutrophil‐mediated clearance of the fungus (Taylor et al. 2007). Research demonstrated that activation of the Dectin‐1 signaling pathway occurs in VVC mice, and treatment with dandelion decoction effectively mitigates fungal infections and inhibits the Dectin‐1 signaling pathway (L. Yang et al. 2018). Another study revealed that leucocephala decoction decreased the expression of NLRP3 proteins and inflammatory cytokines in serum and suppressed ROS release, consequently inhibiting the activation of NLRP3 inflammasomes in VVC treatment (K. Hu et al. 2023). Another study by Zhang and Zhang (2012), underscored the efficacy of LXD in a mouse model of *Candida* vaginitis by demonstrating a reduction in inflammatory damage and inflammatory cytokines, and its mode of action may be connected to an inhibition of the TLR/MyD88 pathway, which may contribute to a reduction in the NLRP3 inflammasome (Bär et al. 2014). These TCM components have positive impacts on inflammatory diseases.

Moreover, berberine is the most abundant bioactive component observed in the traditional Chinese herb, *C. chinensis* Franch. Berberine plays a key role in preventing *C. albicans* from adhering to vaginal epithelial cells through a reduction in neutrophil recruitment to the NLRP3 inflammasome by inhibiting endothelin‐converting enzyme 1 (ECE1) and mucin expression. *ECE1*, a gene specific to *C. albicans* mycelium, is responsible for both mycelial elongation and candidalysin production in the mycelial state. Candidalysin plays an important role in VVC by recruiting neutrophils and activating the NLRP3 inflammasome (Kasper et al. 2018). Berberine inhibits fluconazole‐resistant *C. albicans* by targeting the high‐osmolarity glycerol MAPK pathway, ultimately heightening the susceptibility of fungi to fluconazole post‐treatment (X. Huang et al. 2021). Interventions utilizing berberine prompted the disintegration of the entire biofilm structure, leading to a considerable decrease in the expression of key regulatory genes responsible for biofilm formation, thereby restraining the growth and propagation of mycelial cells (X. Huang et al. 2020).

## 4 *Trichomonas vaginalis* (TV)

### 4.1 TV Pathogenesis

*T. vaginalis*, the etiological agent of trichomoniasis, can infect the female genital tract and cause vaginitis (Hashemi et al. 2021). Trichomoniasis involves damp‐heat affliction, liver‐heat accumulation, and liver and kidney deficiencies, with patients exhibiting characteristic symptoms such as external genital itching, the presence of an abnormal vaginal discharge with a foul odor, and the appearance of foamy, yellowish‐white, thin secretions (Y. Zhang et al. 2022). This microorganism thrives in a moist environment with pH values ranging from 5.2 to 6.6 and a temperature range of 25°C–30°C. Once *Trichomonas* infiltrates the vagina, it feeds on and absorbs the sugar substances within vaginal epithelial cells, effectively consuming lactobacilli and thereby impeding the formation of lactic acid (Haya 2014). *Trichomonas* can adhere to vaginal epithelial cells, inducing apoptosis in these cells via cysteine proteins and leading to the production of IL‐8, which may be a secondary outcome of its cytolytic effect on cells, and the early release of the proinflammatory cytokines, TNF‐α and IL‐1, from damaged epithelial cells, culminating in localized inflammation (Sommer et al. 2005). The resultant compromise of the vaginal immune defense system can render a woman susceptible to infection by various bacteria, and recurrence is likely to occur without proper treatment (Fichorova et al. 2006).

### 4.2 Herbal Medicines for TV

Metronidazole and tinidazole demonstrate a high cure rate for TV; however, their long‐term or high‐dose usage often leads to side effects, such as headache, dry mouth, metallic taste, glossitis, and urticaria, reducing patient compliance and potentially leading to treatment failure (Dai et al. 2016). Effective TCM treatment of TV emphasizes detoxification and the elimination of dampness to address both the underlying cause and its symptoms, aiming to restore the balance of yin and yang. Internally, remedies like Rhizoma Smilacis Glabrae, Zhibai Dihuang pills, and LXD work to clear heat and detoxify, alleviating inflammatory reactions. Moreover, traditional Chinese medicinal agents like Cnidii Fructus, *S. flavescens*, *Taraxacum officinale*, and *P. chinensis cortex*, when directly applied to the affected area, can improve local congestion, inhibit parasite adhesion, and exhibit antibacterial and insecticidal effects (Huo et al. 2023). Du et al. (2002) used a boiled TCM formula comprised of red bean, stem root, and *Euphorbia helioscopia* for fumigating the external genitalia of patients with *T. vaginalis* infection. In addition, *S. flavescens* gel, featuring matrine and oxymatrine, has multifaceted pharmacological effects, including antibacterial, anti‐inflammatory, antiviral, and antitumor properties (Cao and He 2020). Sophora suppositories demonstrate potent insecticidal and antibacterial effects, alleviate vaginal dryness, enhance *Lactobacillus* growth, and boast higher safety. These approaches offer a personalized treatment that addresses both the root cause and the symptoms of TV, presenting a breakthrough in vaginitis treatment.

The combination of oral and topical applications of TCM and Western medicines offers enhanced efficacy compared with using Western medicine alone for the treatment of TV. LXD and Wumei Yinchen Decoction can ease inflammatory reactions when coupled with sitz bath formulations containing *Scutellaria baicalensis*, *C. chinensis*, *Phellodendron chinense*, *Rheum officinale*, and *G. scabra*, which exhibit inhibition against TV pathogens (Du et al. 2002). Certain compounds, such as *C. chinensis*, *Sophora subprostrata*, Semen Strychni, *Mentha haplocalyx*, and *Centella asiatica*, demonstrate significant antitrichomonal effects (Zhou and Qu 2009). Moreover, research has suggested that several combination therapies, including oral metronidazole with a *S. subprostrata* and *P. chinense* decoction for external washing, a benzoic acid metronidazole dispersible tablet with Semen Strychni fumigation, and a Chinese herbal sitz bath (like with *S. subprostrata*, cypress tree, peanuts, Semen Strychni, *Taraxacum*, clover, *Gentianae* and white fresh bark) with metronidazole, offer insecticidal properties, itching relief, and heat‐clearing and dampness‐eliminating effects, surpassing individual drug treatments in efficacy.

## 5 TCM Treatments for Other Types of Vaginitis

### 5.1 Viral Vaginitis

Other common forms of vaginitis include viral vaginitis, senile vaginitis, infantile vaginitis, and mixed vaginitis. Viral vaginitis is caused by infection with viruses, including the herpes simplex virus (HSV), which is mainly transmitted to the genital mucosa through sexual activity. Numerous studies have demonstrated that *Prunella* has a therapeutic effect on HSV‐2 vaginal infections, exerting its anti‐HSV effect through the inhibition of viral penetration into Vero cells (Y. Zhang et al. 2007). LXD is an effective antiviral TCM that has been shown to inhibit genital herpes caused by HSV‐2 by promoting the expression of proinflammatory factors in the blood, such as IL‐6, IL‐10, IL‐12, IFN‐α, and IFN‐γ (Kuang et al. 2018). In addition, “Jie Ze No. 1” (JZ‐1), based on the Qing Dynasty prescription, Yihuang Tang, and modified by modern TCM research, is comprised of various herbs with heat‐clearing, detoxifying, and immune‐enhancing properties, including yellow cypress, *Ginkgo biloba* (ginkgo), nightshade, dandelion, Thlaspi, white skin, Tuckahoe, peony, mint, and borneol (Lin et al. 2019). Duan et al. (2020) built a viral vaginitis model by infecting human vaginal epithelial cells (VK2/E6E7) with HSV‐2 and treating them with JZ‐1 to explore its antiviral effects. They reported that the antiviral effects of JZ‐1 were superior to penciclovir and berberine due to an enhancement of host cell defense and impedance of HSV‐2 adhesion and penetration.

### 5.2 Senile Vaginitis

In postmenopausal women, declining ovarian function and decreasing endogenous estrogen secretion lead to an increase in the incidence of senile vaginitis (Weber et al. 2015). Zhibai Dihuang pills are commonly used to treat senile vaginitis and have been shown to have significantly better therapeutic outcomes than metronidazole alone (Xiao 2018). In addition, the loss of estrogen‐dependent cells and the gradual pathological changes of the vagina and other estrogen‐dependent tissues lead to vaginal atrophy, a thinning of the vaginal mucosa, decreased elasticity of connective tissue, and a decreased defense ability (Y. Sun et al. 2022). Genistein, a natural isoflavone compound found in soy and soy products, can be used as a natural replacement for phytoestrogen and isoflavone (Yu et al. 2021). Genistein enhanced the expression of epidermal growth factor and e‐cadherin in epithelial cells, promoting the proliferation of epithelial cells, increasing the amount of mucosal epithelium and adhesion between cells, and thus thickening the vaginal wall and improving atrophy (Y. Sun et al. 2022).

### 5.3 Infantile Vaginitis

Infantile vaginitis, caused by pathogenic bacteria like *Streptococcus pyogenes* and *Haemophilus influenzae*, leads to poor vulvar development, low sex hormone levels, higher pH levels, and inadequate hygiene in children (Beyitler and Kavukcu 2017). TCM can create an acidic environment that neutralizes toxins and activates the immune system. For example, Jie Tong Yin lotion can be applied externally to inhibit bacteria, alleviate local irritation, and improve immunity (H. L. Liu et al. 2002). A clinical study showed that Jingdaining capsules have higher cure rates and lower recurrence rates than those in a control group who did not receive Jingdaining capsule treatment (Y. F. Yang and Zhou 2008). These TCM strategies exhibit the positive effect of clearing leucorrhea to restore the vaginal environment in children.

### 5.4 Mixed Vaginitis

Mixed vaginitis is characterized by concurrent vaginal infections caused by at least two different pathogens, with lower eradication rates and higher relapse rates than for vaginitis caused by a single pathogen (Tumietto et al. 2019). Treating mixed infections often involves combining an anti‐infective Western drug with TCM to maintain the vaginal microbial balance (Qi et al. 2021). Baicao Fuyangqing suppositories can help restore normal vaginal microbiota by inhibiting harmful bacterial growth, protecting and promoting beneficial bacteria like lactic acid‐producing bacteria, repairing damaged vaginal epithelium, and reducing inflammation (Q. Wang et al. 2023). However, given the overlap between the signs and symptoms of various types of vaginitis, it is difficult to draw firm conclusions. The therapeutic strategies for the alleviation of symptoms and the recovery from a disturbed to healthy *Lactobacillus*‐dominated vaginal flora need further exploration and confirmation in larger trials.

## 6 Conclusions

Vaginitis, characterized by uncomfortable symptoms, such as an abnormal vaginal discharge, itching, irritation, and an unpleasant odor, is a common condition that significantly impacts the quality of life of women. In Western medicine, systemic treatment typically involves oral estrogen, antibiotics like metronidazole or tinidazole, and local medications, such as metronidazole or tinidazole suppositories. TCM has emerged as a pivotal player in ameliorating the clinical symptoms and curbing the recurrence rates of vaginitis. TCM treatments exhibit a reduction in the incidence of adverse reactions and demonstrate commendable clinical outcomes due to their anti‐inflammatory and antibacterial effects on vaginitis. A synergistic approach involving oral Chinese medicine, external Chinese medicine, and integration with Western medicine has been observed to have enhanced therapeutic outcomes. These interventions effectively control the growth of vaginal bacteria, promoting patient recovery and reducing recurrence rates.

Bacterial vaginitis has a complex etiology, with the vaginal microenvironment playing a key role in the disease. While the Chinese herbal medicine compound YHD can regulate the function of various immune cells, Bazhengsan can selectively inhibit harmful bacteria, Cortex *P. chinensis* can inhibit apoptosis, and Shajiziyou can inhibit bacteria and promote tissue regeneration at the same time. The synergistic effects of these different drug components complement each other, thus restoring the acidic state of the vaginal microenvironment.

*Candida* is a common colonizing fungus in the human body; therefore, recurrence of *Candida* vaginitis is common, usually because the initial treatment disrupts the normal growth of vaginal flora. Chinese medicine compounds can be used to inhibit bacteria, restore the vitality of epithelial cells, and enhance local immunity for a gentler and more comprehensive treatment of *Candida* vaginitis, reducing the rate of recurrence.

The inflammatory reaction caused by *Trichomonas* infection necessitates a search for remedies that can both kill the parasite and stop itching. The Western medicine approach to TV primarily utilizes drugs commonly used in the treatment of vaginitis, including metronidazole, which can lead to many side effects in patients. However, the Chinese medicine compound, Wumei Yinchen Decoction, can simultaneously regulate the patient's vaginal pH and kill the parasite, thus removing the cause of the disease and inhibiting inflammation.

The prevalence of viral vaginitis is not high; however, its treatment and management are complex. Chinese herbal compounds have an antiviral effect and can promote recovery from inflammation by improving the body's immunity. For example, JZ‐1 plays a key therapeutic role in treating viral vaginitis through multiple pathways. Herbal ingredients with estrogen‐like effects (e.g., isoflavones, such as chromogranin) can have very good therapeutic effects on geriatric vaginitis, which is caused by estrogen deficiency. Additionally, because of the synergistic properties of its components, the regulation of the body through Chinese medicine is more gentle, which is more appropriate for older adults who have lower pathogen resistance and a lower overall quality of the body.

Similarly, infants are susceptible to vaginal inflammation because of their low pathogen resistance, which is caused by incomplete development of the immune system, and low hormonal levels caused by incomplete development of the gonadal axial nervous system. Compound Chinese medicines can restore the pH balance in the vagina of infants in a nonirritating and safe manner, such as with Jie Tong Yin lotion.

**Figure 3:**
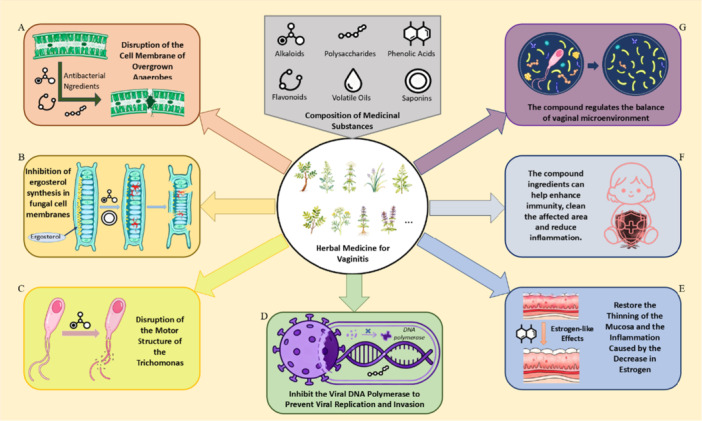
Mechanism diagram of the effect of herbal medicine on vaginitis. Herbal medicines mainly combat vaginitis by (A) inhibiting bacteria, (B) inhibiting fungi, (C) acting on the cilia of *Trichomonas*, (D) inhibiting virus replication, (E) repairing the mucosa, (F) enhancing immunity, and (G) maintaining the microecological balance.

Mixed vaginitis has a mixed etiology and leads to disruptions in the microbial balance, both characteristics that reflect the advantages of TCM. Bai Cao Gynecological Inflammation Clearing Suppository plays an important therapeutic role in the treatment of mixed vaginitis because of its antibacterial properties, its ability to improve immune function, and its estrogen‐like effects (Figure 3 and Table 1). However, more research needs to be conducted related to this substance.

**Table 1:** Herbal treatments for different types of vaginitis.

Types of vaginitis	TCM treatments	Key active components	Proposed mechanisms	Clinical outcomes
Bacterial vaginosis	(1) *Oral TCM:* Yihuang Decoction, Liuwei Dihuang Decoction (LDD), Zhibai Dihuang Decoction (ZDD), Longdan Xiegan Decoction (LXD). (2) *Topical TCM:* Cortex *Phellodendri chinensis* (oral/topical), Bazhengsan, Fufang Furong Effervescent Suppository, compound sea buckthorn seed oil suppository. (3) *Integrated TCM–WM:* LDD + tinidazole, ZDD + metronidazole, LXD + Western medicine, Sophora alkaloid gel + nifuratel capsules, metronidazole + Sophora‐containing lotion	*Dioscorea* polysaccharides, *Atractylodes macrocephala* polysaccharides, *Plantago* seed polysaccharides, *Radix Rehmanniae* (rehmapicrogenin), berberine, *Dianthus superbus*, *Glycyrrhiza*, matrine, sea buckthorn flavonoids, total sea buckthorn polyphenols	(1) *Immune Regulation:* Modulates the functions of T cells, macrophages, and dendritic cells; activates NF‐κB, Jak‐STAT, and MAPK pathways. (2) *Bacteriostasis and Bactericidal Activity:* Inhibits the growth of aerobic/anaerobic bacteria, disrupts bacterial biofilms, and does not harm lactobacilli. (3) *Microenvironment Improvement:* Regulates vaginal pH to restore an acidic environment. (4) *Anti‐inflammatory and Antioxidant:* Reduces oxidative stress and decreases inflammatory factors, such as IL‐4 and IL‐12	(1) *TCM Alone:* Bazhengsan has a lower recurrence rate than metronidazole; Cortex *P. chinensis* improves vaginal epithelial apoptosis and relieves symptoms. (2) Integrated TCM–WM: LDD increases the effective rate of tinidazole from 84.85% to 96.97%; ZDD raises the effective rate of metronidazole from 78.95% to 94.59% with a 3‐month recurrence rate of only 10% (22.5% in the control group); LXD combined with Western medicine achieves a total effective rate of 96.88% (84.88% in the control group)
Vulvovaginal candidiasis	(1) *Topical TCM:* Perillaldehyde, Baofukang Suppository, compound Huoxiang lotion, Honghe lotion (combined with miconazole). (2) *Oral TCM:* Baitouweng Decoction, dandelion decoction, Leucaena decoction, LXD	Perillaldehyde, *Pulsatilla* saponins (pentacyclic triterpenoid saponins), dioscin, berberine	(1) *Antifungal Activity:* Inhibits *Candida* adhesion, hyphal/biofilm formation, and disrupts fungal cell membranes. (2) *Immune Regulation:* Enhances Th1 cell immunity and inhibits Dectin‐1 and TLR/MyD88 pathways. (3) *Anti‐inflammatory:* Reduces inflammatory factors, such as TNF‐α and IL‐1β, and inhibits NLRP3 inflammasome. (4) *Tissue Protection:* Restores the morphology and vitality of vaginal epithelial cells	(1) *TCM Alone:* Baitouweng Decoction group has a recurrence rate of 25% (47% in the fluconazole control group); Baofukang Suppository effectively relieves itching and abnormal discharge. (2) *Combined Medication:* Miconazole + Honghe lotion shows a better cure rate and fungal negative conversion rate than miconazole alone; Huoxiang lotion rapidly alleviates symptoms related to fungal infections
*Trichomonas vaginalis*	(1) *Oral TCM:* Rhizoma Smilacis Glabrae, Zhibai Dihuang pills, LXD, Wumei Yinchen Decoction. (2) *Topical TCM:* Cnidii Fructus, *Sophora flavescens*, *Taraxacum officinale*, Cortex *P. chinensis*, Sophora gel, Sophora Suppository, TCM fumigation (adzuki bean + stem root + *Euphorbia helioscopia*). (3) *Integrated TCM–WM:* LXD/Wumei Yinchen Decoction + Sitz Bath Formula (含 *Scutellaria baicalensis*, *Coptis chinensis*, etc.), metronidazole + *Sophora subprostrata* and *Phellodendron chinense* lotion for external washing	Matrine, oxymatrine, *C. chinensis*, Strychnos Nux‐vomica, *Mentha haplocalyx*, *Centella asiatica*	(1) *Trichomonocidal and Bacteriostatic:* Inhibits *Trichomonas* adhesion and growth, and kills *Trichomonas*. (2) *Anti‐inflammatory:* Reduces inflammatory responses and improves local congestion. (3) *Microenvironment Regulation:* Adjusts vaginal pH and promotes lactobacilli growth	(1) *Integrated TCM–WM:* Metronidazole + Sophora‐containing lotion has better long‐term effects than metronidazole alone; Sophora alkaloid gel + nifuratel capsules achieves a high total effective rate and reduces inflammatory factor levels. (2) *Topical TCM:* Sophora suppositories have high safety, relieve vaginal dryness, and reduce side effects (e.g., headache and metallic taste caused by metronidazole)
Viral vaginitis	(1) *Topical TCM: Prunella vulgaris*, “Jie Ze No. 1” (JZ‐1). (2) *Oral TCM:* LXD	Active components of *Prunella vulgaris* (lignin‐carbohydrate complex), *P. chinense*, *Ginkgo biloba*, *Solanum nigrum*, *T. officinale*, *Thlaspi arvense*, *Dictamnus dasycarpus*, *Poria cocos*, *Paeonia suffruticosa*, *M. haplocalyx*, borneol	(1) *Antiviral:* Inhibits HSV‐2 invasion of host cells and prevents viral adhesion and penetration. (2) *Immune Regulation:* Enhances host cell defense and promotes the expression of proinflammatory factors, such as IL‐6 and IFN‐γ. (3) *Anti‐inflammatory:* Facilitates inflammatory recovery	(1) *TCM Alone:* “Jie Ze No. 1” has better antiviral effects than penciclovir and berberine; LXD alleviates symptoms of genital herpes caused by HSV‐2. (2) *Prunella vulgaris* effectively inhibits HSV‐2 vaginal infection and reduces viral load
Senile vaginitis	*Oral TCM:* Zhibai Dihuang pills. *Topical/Oral:* Genistein (extracted from soybeans and soy products)	Active components of Zhibai Dihuang pills, genistein (isoflavone)	(1) *Estrogen‐like Effect:* Regulates estrogen levels and improves symptoms related to estrogen deficiency. (2) *Tissue Repair:* Promotes epithelial cell proliferation and enhances the expression of epidermal growth factor and E‐cadherin. (3) *Bacteriostatic:* Improves the vaginal microenvironment	(1) Zhibai Dihuang pills show significantly better therapeutic effects than metronidazole alone; genistein thickens the vaginal wall, improves vaginal atrophy, and relieves symptoms, such as dryness and itching. (2) High safety for long‐term use, suitable for elderly patients' tolerance
Infantile vaginitis	*Topical TCM:* Jietongyin lotion. *Oral TCM:* Jingdaining capsules	Active components of Jietongyin lotion, active components of Jingdaining capsules	(1) *Bacteriostatic:* Inhibits the growth of pathogenic bacteria. (2) *Local Protection:* Reduces local irritation. (3) *Immune Regulation:* Enhances immunity and restores vaginal pH balance	(1) Jingdaining capsules group has a higher cure rate and lower recurrence rate (vs. the nonmedicated control group). (2) Jietongyin lotion effectively relieves vulvar irritation symptoms in infants without obvious toxic side effects
Mixed vaginitis	*Topical TCM:* Fufang Furong Effervescent Suppository, Baicao Fuyanqing Suppository. *Integrated TCM–WM:* Anti‐infective Western medicine + TCM	Active components of Fufang Furong Effervescent Suppository, active components of Baicao Fuyanqing Suppository	(1) *Bacteriostatic:* Inhibits various harmful bacteria and protects beneficial bacteria like lactobacilli. (2) *Tissue Repair:* Repairs damaged vaginal epithelium. (3) *Anti‐inflammatory:* Reduces inflammation and maintains microbial balance	(1) Fufang Furong Effervescent Suppository has similar efficacy to clindamycin and is more effective in restoring vaginal flora; Baicao Fuyanqing Suppository improves clinical symptoms and increases the proportion of beneficial bacteria. (2) Integrated TCM–WM improves pathogen clearance rate and reduces recurrence rate (vs. Western medicine alone)

Abbreviations: HSV, herpes simplex virus; IFN, interferon; IL, interleukin; Jak‐STAT, Janus kinase‐signal transducer; MAPK, mitogen‐activated protein kinase; NF‐κB, nuclear factor‐kappa B; TCM, Traditional Chinese Medicine; Th1, Type 1 helper; TLR, toll‐like receptor; TNF, tumor necrosis factor; WM, Western medicine.

## 7 Limitations and Future Perspectives

This review provides a comprehensive summary of the effects, mechanistic insights, and clinical applications of diverse herbal treatments for the distinct types of vaginitis. Herbal medicines for vaginitis emphasize holistic regulation. It employs pattern‐based diagnosis to alleviate local symptoms and reduce recurrence, while also facilitating constitutional regulation to minimize side effects and avoid drug resistance. Particularly suitable for chronic, recurrent cases or patients intolerant to Western medicine, it fundamentally improves the reproductive system's internal environment. However, research on TCM for vaginitis still faces challenges. Current studies are predominantly small‐scale and require deeper mechanistic investigations and larger‐scale trials to enhance the reliability of findings. Additionally, the standardization of herbal formulations needs further advancement to better align with clinical medication guidelines. Further basic research, coupled with more extensive clinical data, is imperative to furnish thorough guidelines for the judicious clinical application of herbal medicines in vaginitis treatment.

## Author Contributions

**Yongming Li:** writing – original draft, investigation, conceptualization. **Huiyu Liu** and **Ruihan Li:** visualization, methodology, editing. **Rong Li:** writing – review and editing, visualization, supervision. **Jiaqi Liu:** conceptualization, writing – review and editing, project administration, supervision.

## Funding

The authors received no specific funding for this work.

## Ethics Statement

The authors have nothing to report.

## Consent

The authors have nothing to report.

## Conflicts of Interest

The authors declare no conflicts of interest.

## Data Availability

The authors have nothing to report.
